# Extended Hormone Profiling Identifies a Wider Network of Duodenal Neuroendocrine Tumor Subtypes

**DOI:** 10.1007/s12022-025-09868-x

**Published:** 2025-06-24

**Authors:** Luvy Delfin, Shereen Ezzat, Sylvia L. Asa

**Affiliations:** 1 Department of Pathology, Institute of Pathology, University Hospitals Cleveland, Case Western Reserve University, 11100 Euclid AvenueRoom 212, Cleveland, OH 44106 USA; 2 Department of Pathology, Emory University, Atlanta, GA USA; 3 Department of Medicine, Endocrine Oncology, University Health Network, University of Toronto, Toronto, ON Canada

**Keywords:** Duodenum, Hormones, Transcription factors, Neuroendocrine tumor

## Abstract

We studied transcription factors and hormones expressed by duodenal neuroendocrine cells in a consecutively diagnosed series of 53 patients with well-differentiated duodenal NETs. There were 30 men; the mean age was 65 years (33 to 81). The study included biopsies (*n* = 18), endoscopic mucosal resections (*n* = 19), and surgical resections (*n* = 16). Three patients had multifocal disease; two had MEN1. Two patients had neurofibromatosis. Metastases were identified in 15/23 patients with biopsied lymph nodes. PAX6 was expressed in 85%, followed by CDX2 in 65%; ARX was expressed in 33%, and no tumors expressed PAX4. The commonest hormone expressed was gastrin; 23 (43%) had diffuse expression, and 12 (23%) had focal reactivity. Pancreatic polypeptide was diffuse and strong in 17 tumors (32%) classified as PP cell NETs; another 3 tumors had focal staining (total *n* = 20, 38%). Serotonin was identified only focally in 14 tumors (26%). Somatostatin was positive in 13 tumors (25%), 3 classical D cell tumors and 10 tumors with focal positivity. PYY was expressed in 10 tumors (19%), diffusely in 1 and focally in 9. CCK was identified in 6 tumors (11%), diffusely in 1 and focally in 5. Staining for glucagon/GLPs, insulin, and motilin was completely negative in all tumors. Thirty tumors (57%) expressed more than one hormone; gastrin was the most frequent. In 2 composite gangliocytoma/NETs (CoGNETs), the NET component expressed PP, and both NET and ganglion cells expressed ARX. These data identify a broad spectrum of duodenal NETs including novel cell types and a high incidence of plurihormonality.

## Introduction

Duodenal neuroendocrine tumors (NETs) are well-differentiated epithelial neuroendocrine neoplasms that develop between the pylorus and the start of the jejunum at the Ligament of Treitz that forms the duodenojejunal flexure. These tumors arise from duodenal mucosa that is rich in neuroendocrine cells of many types; they synthesize and secrete hormones that regulate the digestion and absorption of nutrients (1). Gastrin is key to regulating the acid breakdown of food. Glucagon and glucagon-like peptides (GLPs) are well-known to play a role in glucose metabolism and satiety, but they are also important in intestinal mucosal growth and the regulation of intestinal brush-border enzymes and gastrointestinal motility. Somatostatin, widely recognized as an inhibitory hormone due to its mechanism of action that decreases adenylate cyclase, reduces multiple gastrointestinal actions directly and indirectly by inhibiting other hormones, resulting in decreased gastric acid secretion, gastric emptying, and gut motility, and inhibition of pancreatic glucagon and insulin. Cholecystokinin (CCK) regulates gallbladder contraction and the release of stored bile. Pancreatic polypeptide (PP), peptide YY (PYY), serotonin, and motilin are also produced in the duodenum and are involved in intestinal motility.

Transcription factors are implicated in the development of endocrine cells and in NETs; they can be used to determine the site of origin and cytodifferentiation (2). It has been thought that caudal type homeobox 2 (CDX2) is a reliable biomarker of gastrointestinal origin and indeed is expressed in almost all jejunoileal NETs. SATB2 is expressed by colorectal and terminal ileal NETs (3;4) but also by many other NETs (5;6). The profile of transcription factor expression by duodenal NETs has not been carefully studied. Unlike the jejunum and ileum, where the vast majority of NETs are composed of serotonin-producing EC cells, NETs of the duodenum are a heterogeneous group of tumors. In other body sites, NETs have been subtyped based on the cell of origin and hormone production. The most complex model is the pituitary, where tumor cytogenesis and degree of cell differentiation are more valuable than tumor grade (7). In the rectum and appendix, serotonin-producing EC cell NETs are known to be more aggressive, presenting at larger size and with higher rates of metastasis than L cell NETs that express GLPs, PP, and PYY (8–11). Recently, the pancreas has also been shown to have prognostically relevant NET subtypes based on transcription factor and hormone expression (12). A few papers have attempted to study the expression of hormones and transcription factors in duodenal tumors (13;14). These have focussed mainly on the production of gastrin that can be associated with Zollinger-Ellison syndrome and multiple endocrine neoplasia type 1 (MEN1) syndrome (15–17) or of somatostatin (18) that may be associated with a unique psammomatous tumor in patients with neurofibromatosis (19). An unusual composite tumor composed of a ganglioneuroma and NET, known as CoGNET (formerly called “gangliocytic paraganglioma” but now clearly recognized as a misnomer since there is no paraganglioma component) (20) has also received attention (13;16;21). Apart from these three types, it is generally considered that the remainder are “non-functioning” tumors (13).

Despite the abundance of hormone-secreting cells in the duodenum, there has not been a clear role defined for hormone measurement in assessing the clinical features at presentation of patients with these tumors, nor is it standard of care to perform hormone immunohistochemistry to allow the development of a tumor classification that may provide risk stratification for these patients. Hormones provide helpful circulating biomarkers for surveillance (22), and recurrence may be detected earlier using hormone assays that can improve the management of patients with these neoplasms. In a different setting, when a secondary tumor is identified in the liver or another metastatic site, the localization of a primary tumor often rests on the immunohistochemical hormone and transcription factor profile of the secondary tumor (23); therefore, it is helpful to have a clear understanding of these biomarkers.

We investigated the immunohistochemical profile of a sequential series of duodenal NETs and assessed the clinicopathological correlates of transcription factors and hormone production.

## Materials and Method

### Cohort and Clinicopathologic Characteristics

With institutional research ethics approval, a retrospective review of the pathology files was performed to identify well-differentiated NETs of the duodenum consecutively diagnosed between 2012 and 2022 at the University Hospitals Cleveland Medical Center (UHCMC). Three cases with no tumor block or no residual tumor in the block(s) were excluded. Pathologic variables including Ki67 labeling index, tumor size, and tumor stage according to the AJCC Cancer Staging Manual 8th edition were collected. Clinical, radiological, and biochemical recurrence data were obtained from the clinical chart.

### Immunohistochemistry and Tumor Classification

The slides generated at the time of diagnostic workup were reviewed including H&E-stained slides, immunohistochemical stains for chromogranin and Ki67, and any other stains performed for clinical diagnosis. Stains were performed on all cases to determine the expression of transcription factors implicated in pancreaticoduodenal endocrine cell development including (CDX2, Cell Marque predilute, Roche Ventana stainer), paired box gene 4 (PAX4, Invitrogen PA1-108 1:100, Roche Ventana stainer), paired box gene 6 (PAX6 Invitrogen PA5-25,970, 1:50, Roche Ventana stainer) (24) and aristaless-related homeobox X-linked (ARX Abcam 308,260, 1:100, Roche Ventana stainer) (25), and hormones including gastrin (Cell Marque predilute, Leica Bond stainer), glucagon and GLPs (Cell Marque predilute, Roche Ventana stainer), insulin (Cell Marque predilute, Leica Bond stainer), somatostatin (Cell Marque predilute, Roche Ventana stainer), pancreatic polypeptide (PP, Abcam 1:200, Roche Ventana stainer), peptide (PYY, Abcam 112,474, 1:600, Biocare Intellipath Autostainer), serotonin (Dako, 1:50, Leica Bond stainer), motilin (Invitrogen PA5-64,415, 1:500, Roche Ventana stainer), and cholecystokinin (CCK, Abcam ab134713, 1:50, Roche Ventana stainer). Staining in tumor cells was recorded as negative or positive; the latter was then recorded as strong or weak, diffuse or focal.

### Statistical Analyses

Analysis was performed using IBM SPSS 29®. Comparisons of means for continuous variables were performed using non-parametric tests (Independent samples median test and Mann–Whitney *U*). Proportions of categorical variables were compared using Chi-square and Fisher test as required.

## Results

### Clinical Data

A total of 53 well-differentiated NETs of the duodenum fulfilled the criteria for inclusion (Table 1). There were 30 men and 23 women; their ages ranged from 33 to 81 years with a mean age of 65 years. The study included biopsies as well as resections that varied from endoscopic mucosal resections to pancreaticoduodenectomy specimens; tumor size was not established for biopsies, but among the 35 resections, the tumors varied from 0.6 to 5.2 cm. Three patients had multifocal disease; one was proven to have MEN1 on genetic testing and one other was thought to have the disorder clinically. Two tumors were in patients with known neurofibromatosis. In 22 patients (41%), lymph node status was determined at the time of the procedure; in 7 patients, all lymph nodes were negative, while the other 15 had from 1 to 6 positive lymph nodes.

**Table 1:** Clinical features of patients with duodenal NETS

Sex	30 men 23 women
Age (years)	33–81 (mean 65)
Specimen	Biopsy 18 EMR 19 Surgical resection 16
Tumor size	0.6 to 5.2 cm
Nodal metastasis	15/22
Outcome	Disease free 30/48 Recurrent disease 13/48 DOD 5/48

Most patients presented with non-specific complaints of gastrointestinal discomfort and abdominal pain. Twelve patients had a clinical diagnosis of Zollinger-Ellison syndrome and had elevated circulating gastrin levels measured. Measurement of circulating hormones was not routinely performed.

Clinical follow-up data were available for 48 patients; the other 5 were lost to follow-up shortly after the procedure. Five patients died of disease within two years of diagnosis. In follow-up periods ranging from 1 to 12 years, 30 patients had no evidence of disease. The remainder were alive with disease (including metastases to liver and bone) at up to 10 years after diagnosis. Interestingly, 11 patients had multiple neoplasms; in addition to those with MEN1 and neurofibromatosis, there were 4 breast carcinomas, 3 colon adenocarcinomas, 2 prostate carcinomas, and one myeloma. One patient with multiple primaries had a germline CHEK2 pathogenic variant.

### Morphologic Findings

The tumors consisted of nests and cords of well-differentiated neuroendocrine cells arising in the mucosa and infiltrating through the submucosa, muscularis propria, and beyond. The majority had focal pseudoglandular formations. The tumor cells were generally polygonal with poorly defined cell borders; in some tumors, they were elongated and almost spindle-shaped. The tumor cell nuclei were relatively uniform, bland, and round with salt-and-pepper chromatin. Some tumors had stromal fibrosis of varying degrees. Two had psammoma bodies scattered throughout; calcifications could be seen within tumor cells undergoing necrosis, and none were associated with papillary structures. Two tumors had a coexisting ganglioneuroma component and were classified as CoGNETs.

### Immunohistochemical Findings

All tumors in this study had been examined for the expression of neuroendocrine markers including INSM1, synaptophysin, chromogranin, and/or somatostatin receptor 2 (SSTR2), and keratins (AE1/AE3, CAM5.2) for clinical diagnosis. Not all were performed in every case, but all tumors in this study had expression of chromogranin and at least one keratin. The Ki67 labeling index was used to grade all tumors; 31 were G1, and 2 were G3, while the rest were G2.

**Fig. 1:**
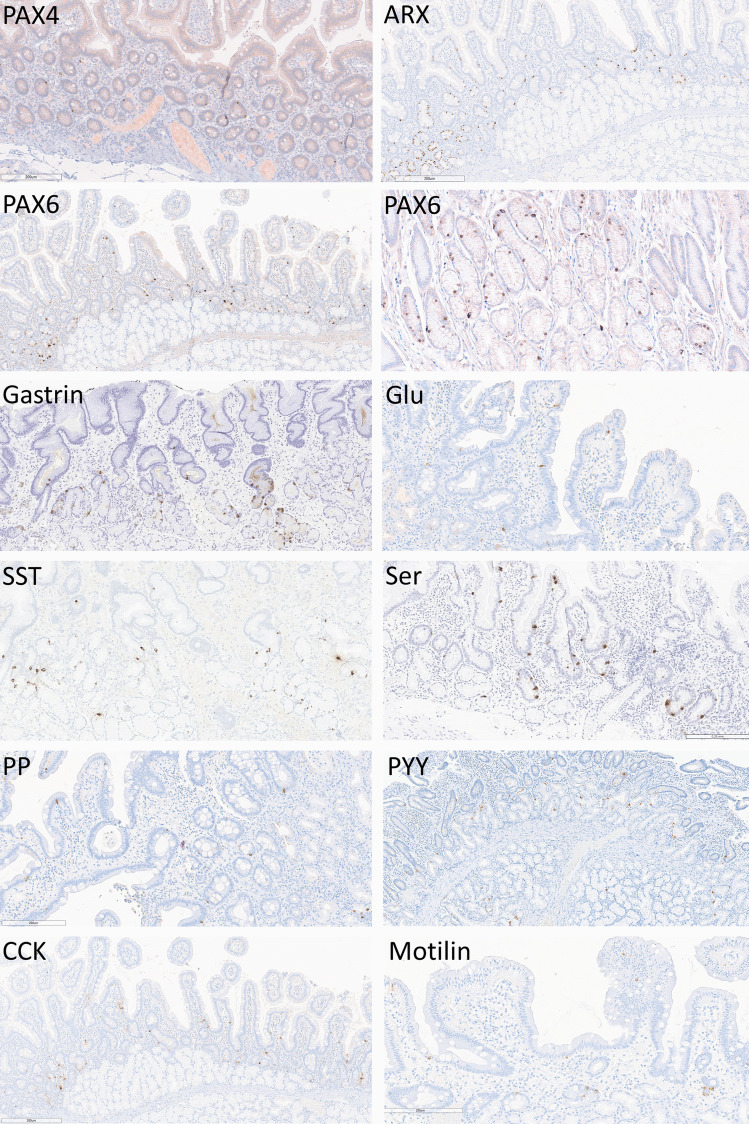
Transcription factor and hormone expression in the nontumorus duodenum. Scattered cells express PAX4, ARX, and PAX6 in the nucleus and have cytoplasmic positivity for Gastrin, Glucagon/GLPs (GLU), Somatostatin (SST), Serotonin (Ser), Pancreatic Polypeptide (PP), Peptide YY (PYY), Cholecystokinin (CCK) and Motilin

The pattern of expression of the various transcription factors and hormones in nontumorous duodenum is shown in Fig. 1. The targets of this study are identified in endocrine cells within the epithelium of the duodenum with variable numbers, all scattered throughout the nontumorous mucosa. The expression of CDX2 could not be established clearly in endocrine cells due to the intense and abundant positivity in the nontumorous epithelium that predominates in the normal mucosa.

**Fig. 2:**
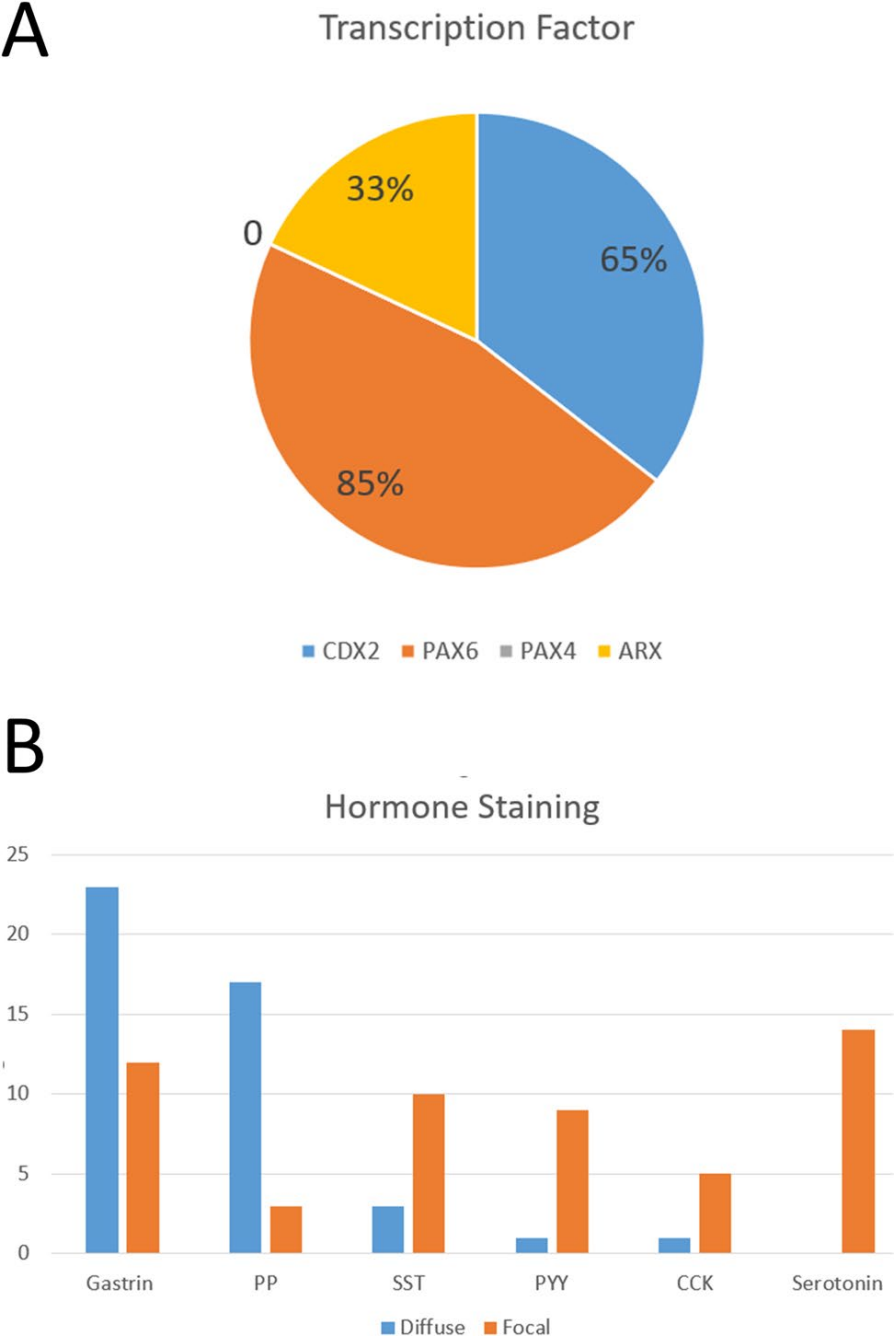
Transcription factor and hormone expression in duodenal neuroendocrine tumors (NETs). **A** PAX6 is expressed by 85% of these tumors, CDX2 by 65%, and ARX by 33%; PAX4 was not expressed in any of the tumors. **B** Gastrin was the most frequent hormone detected, with diffuse positivity in 23 tumors and focal staining in 12. Pancreatic polypeptide (PP) reactivity was diffuse in 17 and focal in 3. Somatostatin (SST) was diffusely positive in 3 tumors and focal in 17. Peptide YY (PYY) and cholecystokinin (CCK) were each diffuse and strong only in 1 tumor each, but were focally positive in 9 and 5 tumors, respectively. Serotonin was not diffusely positive in any tumor but was focally expressed in 14 tumors

**Fig. 3:**
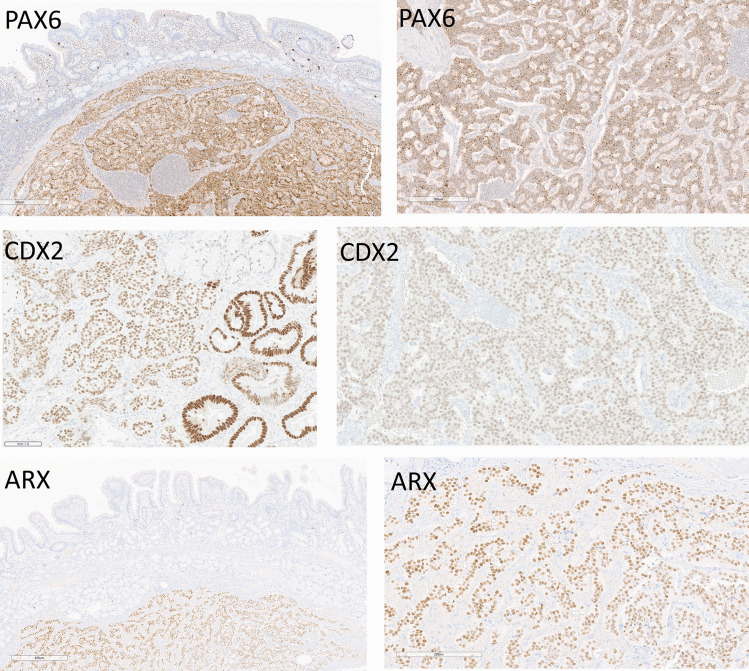
Transcription factor expression in duodenal neuroendocrine tumors (NETs). Nuclear PAX6 (top left and right) was the most common finding in these tumors. CDX2 staining was less intense than in the nontumorous epithelium (middle left) and generally was weak in the tumors (middle right). ARX was usually strong in the tumors where it was expressed (bottom left and right)

Transcription factor expression is summarized in Fig. 2a. In the tumors examined, the most commonly expressed transcription factor was PAX6 that was expressed in 85% of the tumors (Fig. 3 top). CDX2 was expressed by only 65% of the tumors, and in half of those, the staining was weak (Fig. 3 middle). ARX expression was positive and strong in 33% of the tumors (Fig. 3 bottom). Staining for PAX4 was negative in all tumors.

**Fig. 4:**
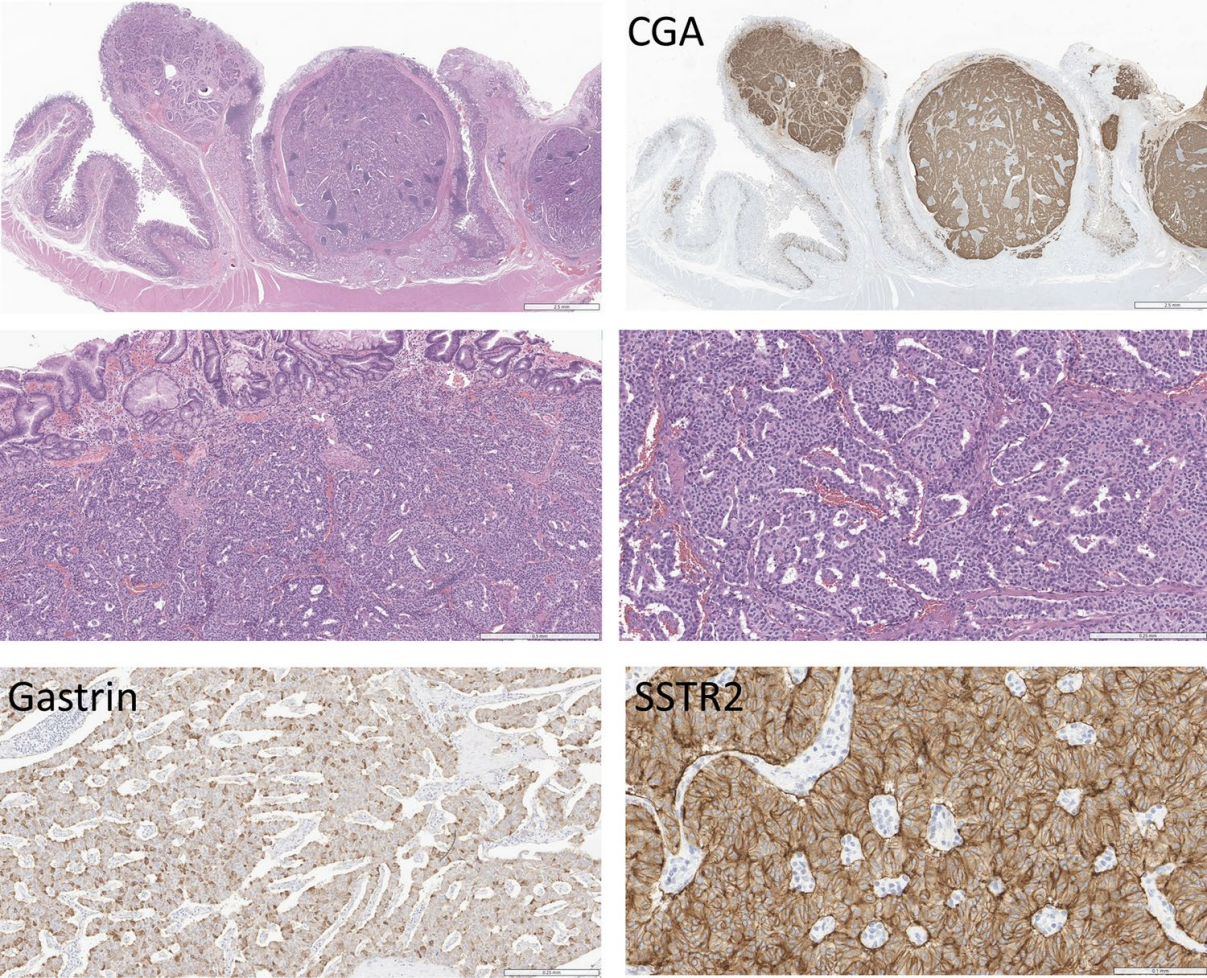
Multifocal duodenal G cell neuroendocrine tumor (NET). The duodenum of a patient with MEN1 contains multifocal well-differentiated neuroendocrine tumors (top left) that stain for chromogranin A (CGA, top right). The tumors are composed of nests of epithelial cells with pseudoglandular formations and vascular stroma (middle left and right). The tumor stains strongly and diffusely for gastrin (bottom left) and somatostatin receptor 2 (SSTR2, bottom right)

**Fig. 5:**
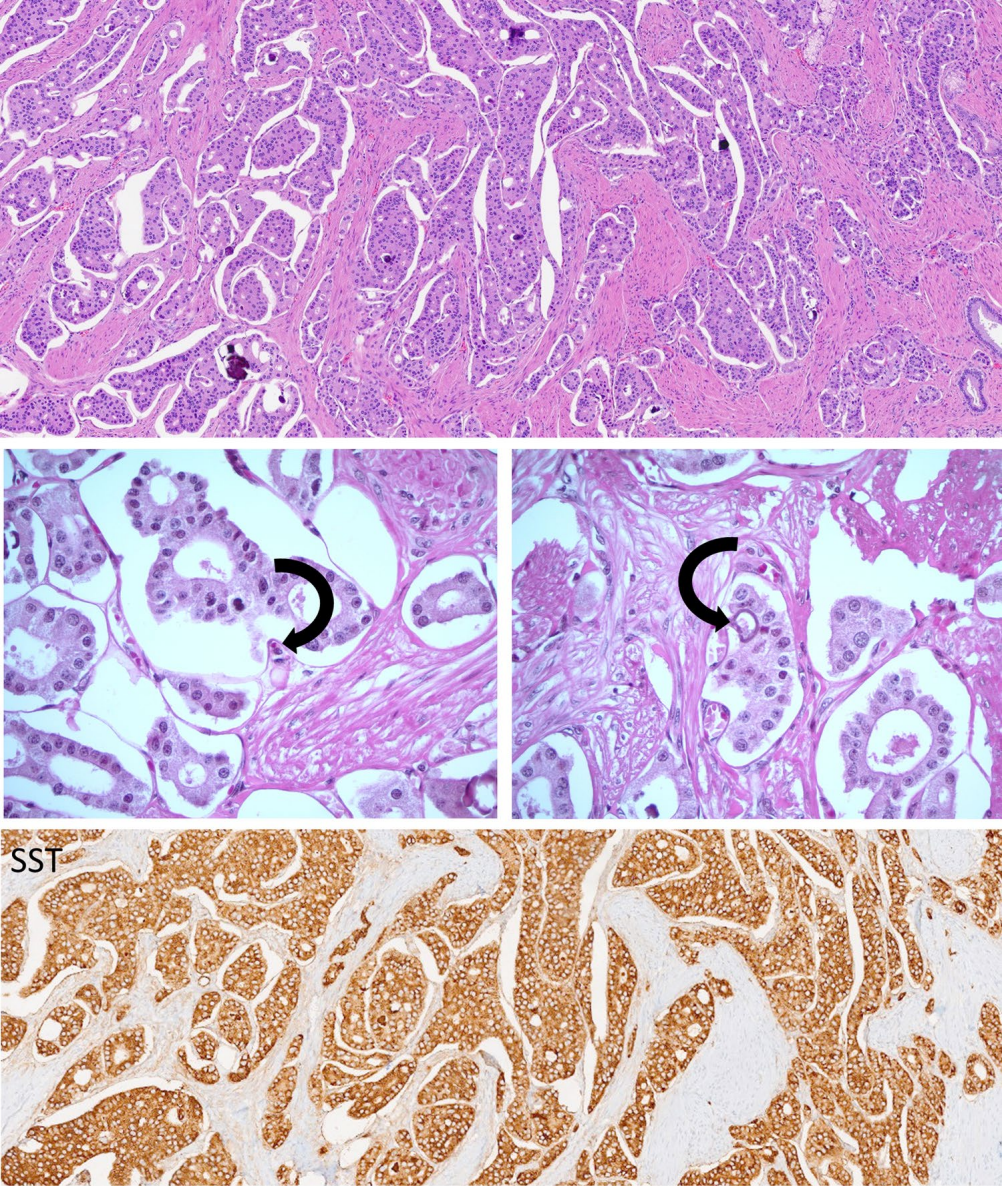
Duodenal D cell neuroendocrine tumor (NET) with psammoma bodies. This tumor is composed of nests and cords of epithelial cells in a relatively fibrous stroma with focal calcifications that form psammoma bodies (top). Psammoma bodies are seen forming within tumor cells (middle left and right, arrows). The tumor is strongly and diffusely positive for somatostatin (bottom)

**Fig. 6:**
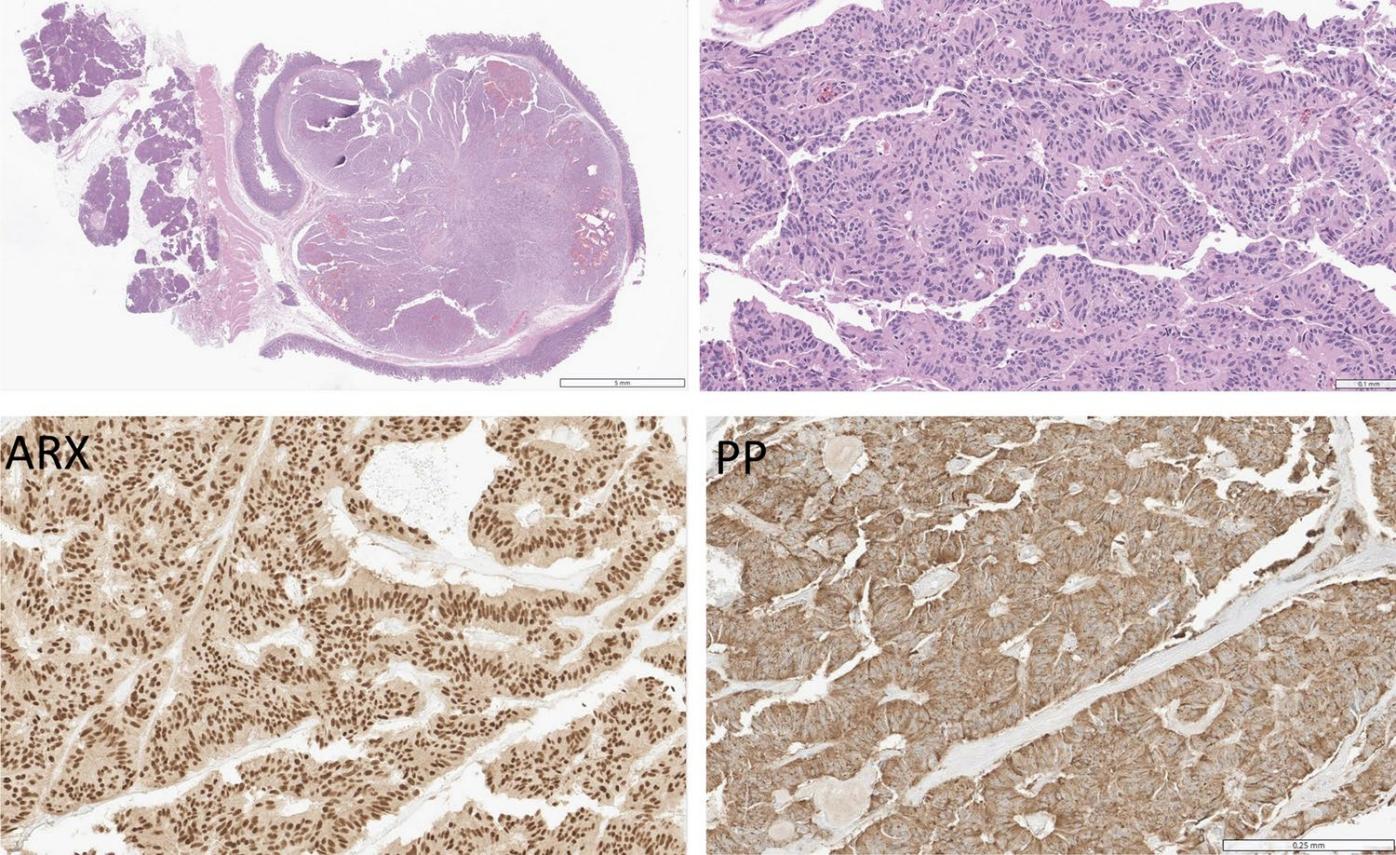
Duodenal PP cell neuroendocrine tumor (NET). This tumor forms a polypoid lesion in the duodenal wall (top left). It has trabecular architecture, and the epithelial cells are elongated and spindle-shaped (top right). The tumor cell nuclei stain for ARX (bottom left) and they have diffuse, strong cytoplasmic positivity for pancreatic polypeptide (PP)

**Fig. 7:**
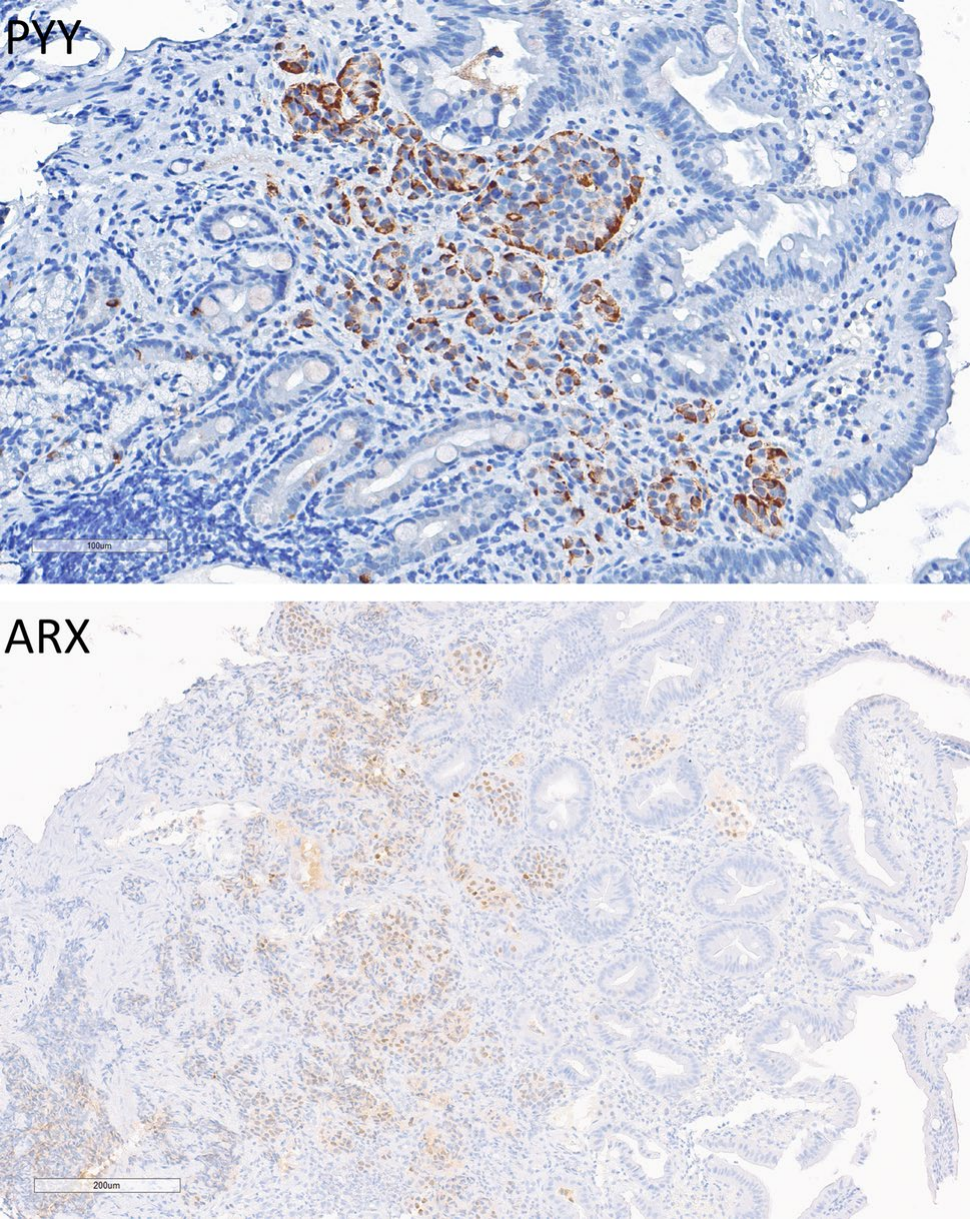
Duodenal PYY cell neuroendocrine tumor (NET). This intramucosal tumor is composed of nests of epithelial cells that express cytoplasmic PYY (top) and nuclear ARX (bottom)

**Fig. 8:**
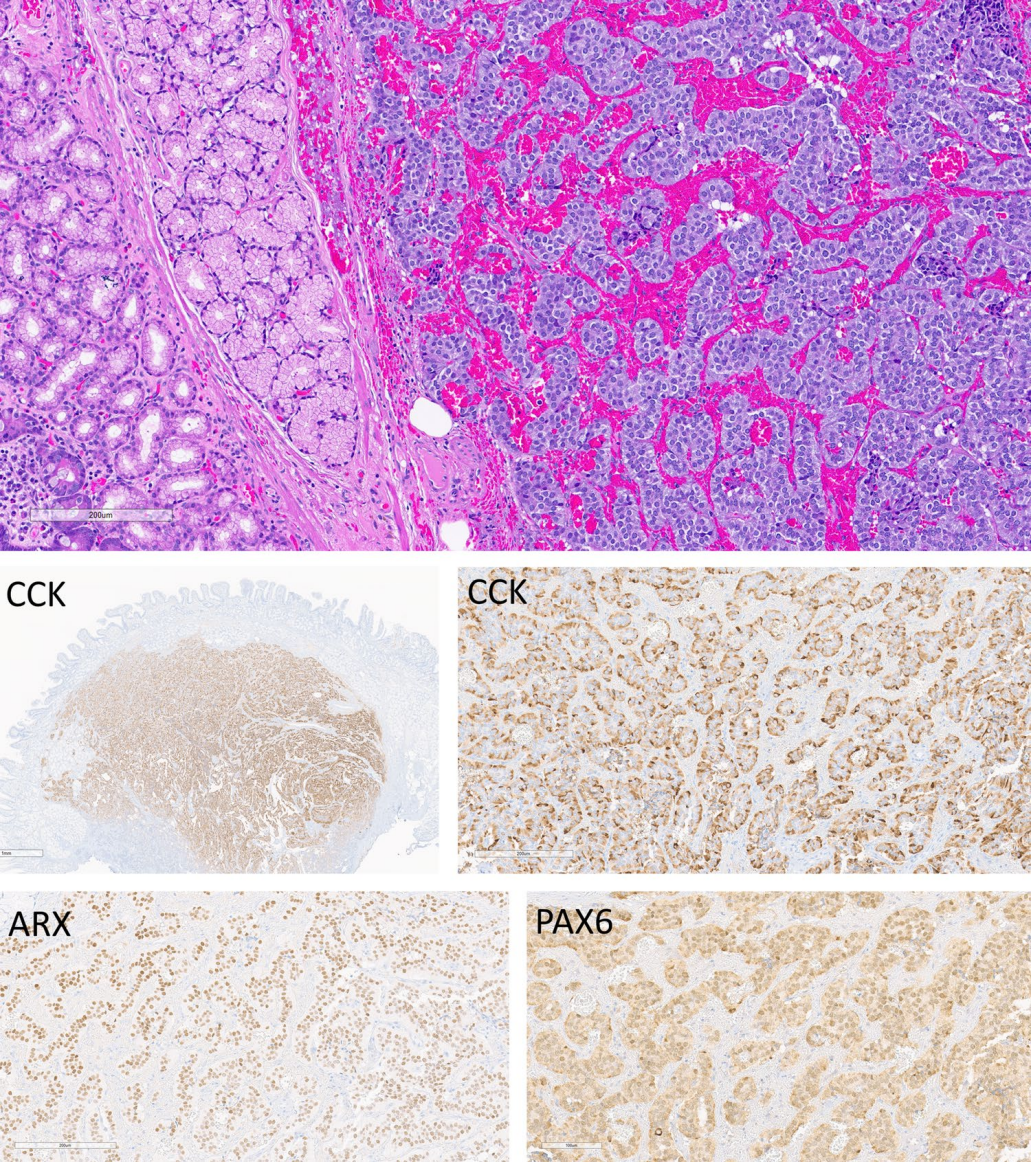
Duodenal CCK cell neuroendocrine tumor (NET). This tumor is composed of nests and cords of epithelial cells in a hemorrhagic stroma (top). The tumor cell cytoplasm stains for CCK strongly and diffusely (middle left and right) and the tumor is also positive for ARX (bottom left) and PAX6 (bottom right)

The results of hormone staining are summarized in Fig. 2b. The most commonly expressed hormone was gastrin. Staining was strong and diffuse in 23 tumors that could be classified as G cell tumors (Fig. 4) and only focal in 12. Thirteen tumors were positive for somatostatin; three of these were strong and diffusely positive, resembling D cells, and two of these had psammoma bodies (Fig. 5); both of these were in patients with known neurofibromatosis. Ten tumors had focal positivity for somatostatin. PP was expressed in 20 tumors, and 17 of those had diffuse strong staining consistent with PP cells (Fig. 6). One tumor expressed PYY diffusely, and 9 others had focal staining (Fig. 7). CCK was expressed in 6 tumors, but only 1 tumor had strong and diffuse positivity (Fig. 8). Serotonin was expressed in 14 tumors, but none had the diffuse positivity seen in ileal EC cell tumors, and this finding was not associated with stromal fibrosis. Staining for glucagon/GLPs, insulin, and motilin was completely negative in all tumors.

**Fig. 9:**
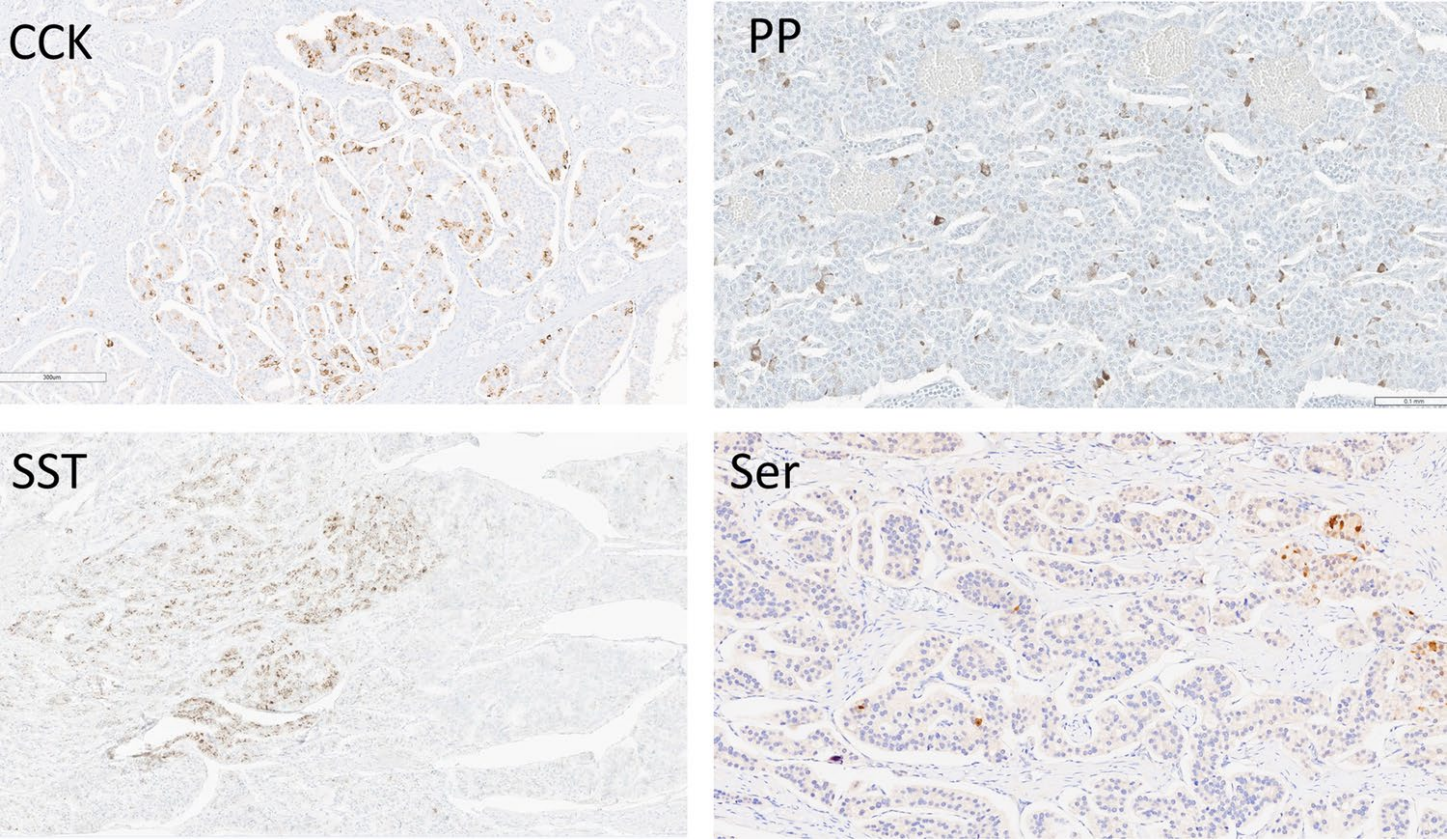
Plurihormonal patterns in duodenal neuroendocrine tumors (NETs). Many of the tumors in this series had expressions of multiple hormones with focal positivity for CCK (top left), PP (top right), somatostatin (SST, bottom left) or serotonin (Ser, bottom right)

An unexpected finding was the common occurrence of plurihormonal patterns in these tumors (Table 2). Gastrin production was unique in only 10 tumors, while 25 all had expression of other hormones, including PP, PYY, and somatostatin. Only 6 tumors had monohormonal SST expression, while 7 were plurihormonal. The one tumor with strong and diffuse positivity for CCK was negative for all other hormones. The majority of tumors producing PP and all the tumors expressing PYY and serotonin were plurihormonal. The pattern of staining of these plurihormonal tumors was usually scattered cells, often in small clusters (Fig. 9). Only 4 tumors in this series were completely negative for all hormones; one of these expressed ARX and one expressed CDX2 weakly.

**Table 2:** Monohormonal versus plurihormonal profiles of duodenal neuroendocrine tumors

Hormone	Total positive	Diffuse	Focal	Plurihormonal
Gastrin	35	23	12	25
PP	20	17	3	18
SST	13	3	10	7
PYY	10	1	9	10
CCK	6	1	5	5
Serotonin	14	0	14	14

There was no correlation between the expression of hormones and the expression of transcription factors.

**Fig. 10:**
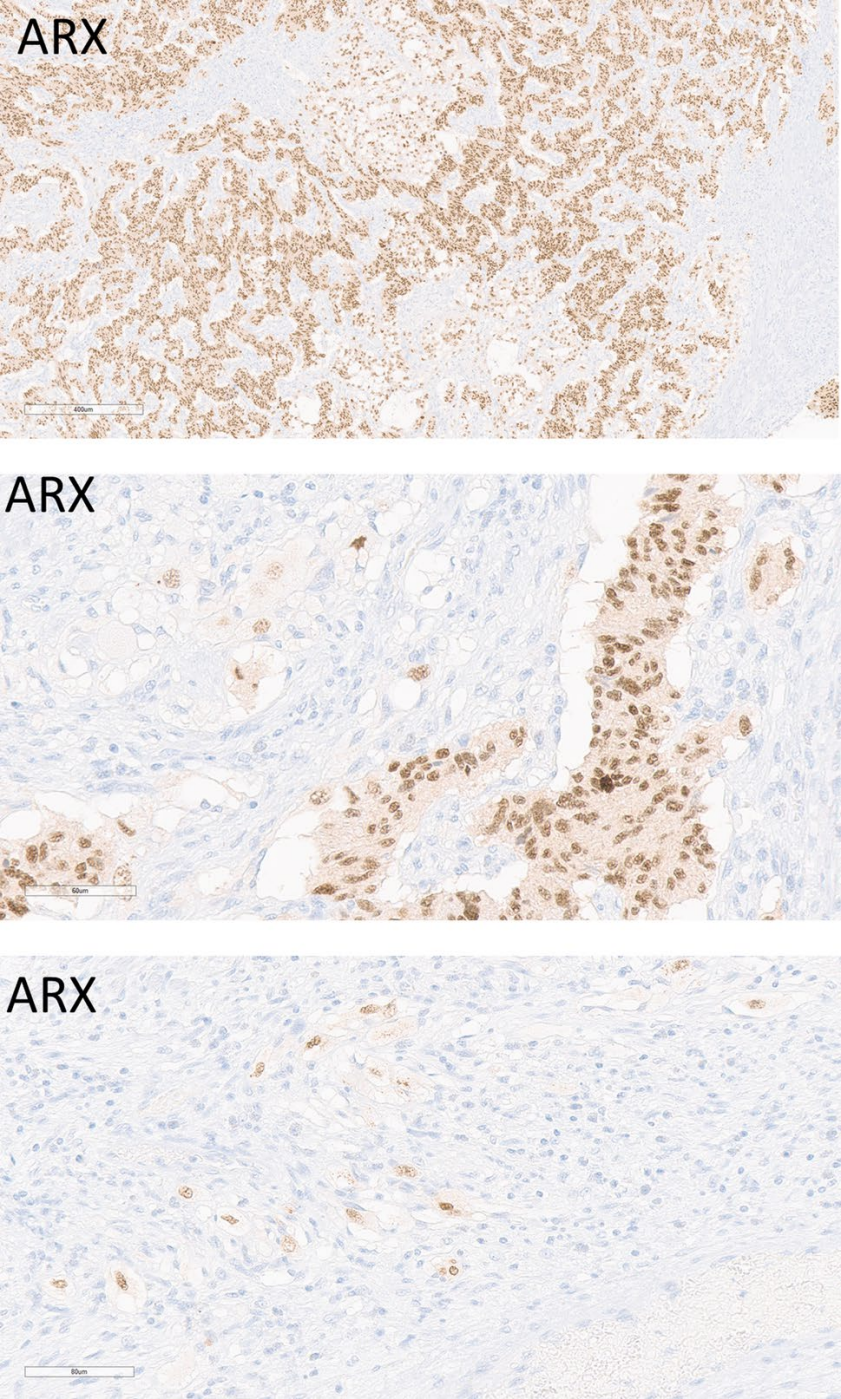
Duodenal Composite Ganglioneuroma and Neuroendocrine Tumor (CoGNET). These tumors express ARX in both the epithelial NET (top and middle) and the ganglion cells of the ganglioneuroma (middle and bottom)

The two CoGNETs offered an interesting opportunity to study the patterns of duodenal neuroendocrine transcription factors and hormones. As reported previously, both expressed PP in the epithelial component (20;21;26)***.*** One also expressed SST. Both were positive for ARX and interestingly, this was also seen in the nuclei of the ganglion cells (Fig. 10).

### Clinical Correlations

The clinical presentation of patients with duodenal NETs was associated with hormone excess in patients who had diffuse gastrin positivity; those patients had gastric ulcerations and other manifestations of gastrin excess. None of the patients with serotonin-producing tumors had clinical evidence of carcinoid syndrome. The patient with a tumor producing CCK had presented with symptoms of cholecystitis and had undergone cholecystectomy. Many patients in this series had type 2 diabetes mellitus, and many complained of cramps, diarrhea, and abdominal pain; it is not possible to attribute those disorders to the production of specific hormones.

Of the 18 patients who died of disease or were alive with recurrent or metastatic disease (Table 3), all but one had a tumor with at least focal positivity for gastrin, and 8 had serotonin reactivity in their tumors. The one tumor that was negative for gastrin was also completely negative for all hormones. Only one tumor stained for gastrin alone; the remainder all had a plurihormonal profile. There was no correlation of adverse outcomes with the Ki67 labeling index; while 9 of them were G2 with Ki67 positivity ranging as high as 15%, the remainder were G1 tumors. Tumor stage was more valuable for prediction; of the 15 patients with lymph node metastases at the time of diagnosis, only 6 are alive without evidence of disease, and 4 of those have less than 24 months of follow-up.

**Table 3:** Immunoprofiles of 18 duodenal neuroendocrine tumors with recurrence, metastasis and/or death of disease

Hormone	Total	Diffuse	Focal
Gastrin	17	8	9
PP	6	3	3
SST	5		5
PYY	6		6
CCK	0		
Serotonin	8		8
None	1		

Expression of serotonin correlated with tumor recurrence/metastasis (*p* = 0.053). Moreover, expression of somatostatin correlated with younger patient age (*p* = 0.028). Conversely, PAX6 reactivity displayed an inverse association with tumor grade (*p* = 0.035) and tumor size (*p* = 0.056).

## Discussion

This is the first comprehensive study of duodenal NETs to determine the role of transcription factor and hormone profiling in the classification of duodenal NETs. While it is not complete in that we have not studied every transcription factor and hormone in the duodenum, we hope that the data will encourage more detailed studies to build on this novel network of duodenal tumor subtypes.

Interestingly, we have found that PAX6 is the best biomarker of these tumors, being expressed in 85% of duodenal NETs. This biomarker has been considered to be specific for pancreatic NETs (27) but the distinction of pancreatic from duodenal tumors cannot rely on this marker. This is not surprising, since it is well documented that deletion of the PAX6 gene not only affects pancreatic A cell development but also eliminates duodenal neuroendocrine cells and gastrin and somatostatin cells of the distal stomach (28). Consistent with this notion, we noted that PAX6 correlated with smaller tumor size and lower grade. What is more confounding is the complete lack of PAX4 immunoreactivity in these tumors, despite its presence in nontumorous duodenal neuroendocrine cells and its important role in their development (28). This may be an explanation for the lack of GLPs in our series of tumors. Both pancreatic and duodenal tumors can express CDX2, but it may be weak or negative as well (12).

ARX has been implicated in the differentiation of pancreatic A cells that produce glucagon (25); however, it should not be surprising that it is also expressed in cells that produce GLPs throughout the gastrointestinal tract. ARX expression has been identified in gut L cells that produce GLP1 and in K cells that produce the related incretin glucose-dependent insulinotropic peptide (GIP, also known as gastric inhibitory peptide) (29). ARX has also been detected in the cells that secrete CCK, secretin, gastrin, and ghrelin, and loss of ARX results in decreased or absent populations of these cells (30). In our study, we identified ARX in 33% of duodenal NETs. In these tumors, it is unassociated with glucagon or GLPs, as these were not expressed in any of the tumors. In addition, ARX expression has been associated with alternative lengthening of telomeres (ALT) and poor prognosis in pancreatic NETs, and while it is mainly expressed in glucagon-producing tumors in that location, it has also been expressed in a subset of insulin-producing pancreatic NETs with poor outcome (31–35); this finding emphasizes the importance of classification of NETs by transcription factor expression that may have prognostic value. It is known that A cell lineage tumors are more susceptible to MEN1 mutations, which in larger tumors are associated with DAXX/ATRX loss, which is associated with ALT. We did not find any prognostic value of ARX in this series, but the number of cases is small, and we did not examine ALT in these tumors.

The transcription factor pancreatic-duodenal homeobox 1 (PDX1) is important in the differentiation and development of the pancreas and duodenum, and it is expressed in a significant number of pancreatic NETs (36;37). While some studies have shown that its expression in tumors is limited to the pancreas and is not found in duodenal tumors (37), others have found it in all duodenal tumors (37;38) and it appears to be less specific than originally claimed, also expressed in other sites (39;40). Because its expression has already been shown not to correlate with hormone production (41), it was a low priority for investigation in this study.

Similarly, the insulin gene enhancer-binding protein islet-1 ISL1 was initially considered to be a pancreatic-specific transcription factor but has subsequently been identified in multiple neuroendocrine and non-endocrine cells and tumors throughout the body (42), and it appears to have no role in hormone specification and cell type development.

Gastrin-producing tumors are the most common duodenal NETs, and most of these are monohormonal, with diffuse positivity that allows them to be classified as G cell tumors. However, more than 40% of gastrin-producing tumors are also plurihormonal, with scattered cells expressing other hormones.

Interestingly, the second most common hormone produced by these tumors is PP. The function and systemic expression of this hormone are poorly understood; while some investigators have called it a “functionless” hormone, it has been speculated to cause clinical symptoms, including diarrhea, and may even be a cause of diabetes (43–49).

Somatostatin-producing D cell tumors are well described and known to have several unique characteristics including the formation of psammoma bodies and association with NF. We also identified frequent focal expression of this hormone in tumors that did not have these classical features. Interestingly, expression of somatostatin was associated with younger age, possibly because of the influence of genetic predisposition in patients with NF.

We identified tumors that produced PYY and CCK diffusely. Serotonin was only found with focal reactivity; none of the positive tumors had the morphology of solid nests composed of basophilic cells as seen in the classical EC cell tumors of the small bowel, nor was there any associated stromal fibrosis that is often a feature of functional tumors causing the carcinoid syndrome. None of our patients was known to have carcinoid syndrome. Expression of this hormone was the only one that came close to having significance in predicting a bad outcome.

Only 4 tumors were negative for hormones; it is possible that these are true “null cell” tumors (7), but it may be that they express hormones which we did not study. For example, we were unable to carry out stains for secretin and GIP that might have been produced by these tumors. The study of GIP may be of particular interest going forward since this product of K cells may be affected by the use of GLP1-agonists in the treatment of diabetes and obesity.

The abundance of plurihormonal tumors in this series has been noted in previous studies (15;16). This finding is uncommon in other sites where there are multiple hormones produced by neuroendocrine cells, such as the pituitary and pancreatic islets. In those tissues, there are clear patterns of hormone production by 6 and 4 cell types, respectively. In the duodenum, it has been postulated that there are multiple cell types (1), but this remains to be proven. It is unknown whether the classical “one cell-one hormone” theory is actually true in the duodenum; it may be that the precursor cells that give rise to these tumors are also plurihormonal. However, it may be that the focal expression of some hormones represents trapped nontumorous tissue; this is not uncommon in other endocrine organs such as the pituitary (50), and given the abundance of endocrine cells in the duodenal mucosa, it cannot be completely excluded, but it is less likely given the linear structure of that organ and the scattered nature of the endocrine cells.

Interestingly, there was no clear correlation between transcription factor expression and hormone production as in other sites. It remains to be determined if there are transcription factors that are unique to any one given cell type.

We did not examine the localization of vasoactive intestinal peptide (VIP) that is expressed in nerves of the duodenum but not in epithelial neuroendocrine cells, nor that of adrenocorticotropic hormone (ACTH), growth hormone (GH), GH-releasing hormone (GHRH), calcitonin, vasopressin, or other hormones that have been expressed ectopically in tumors of the gastroenteropancreatic tract. When they are expressed and give rise to clinical syndromes, these hormones can be localized in the offending tumor, but usually, the expression of these ectopic hormones is an unusual feature that does not impact classification.

A weakness of our study is the lack of clinical data, including measurement of hormones produced by these tumors. Most of the patients presented with what are classified as “nonspecific” gastrointestinal complaints. It is not considered standard of care to measure duodenal hormones in this setting with the exception of gastrin levels in patients with presumed Zollinger-Ellison syndrome. One of the main messages of our study is that there is a need for more awareness of the role of hormone evaluation in the assessment of patients with duodenal NETs.

The management of patients with duodenal NETs varies from surveillance to endoscopic submucosal resection to complete resection by gastro-duodenectomy or Whipple procedure. Our study has limited outcome data due to the small number of cases, but it identifies the importance of gastrin that was expressed in all but one tumor with an adverse outcome; moreover, all but one of these more aggressive tumors had a plurihormonal profile. Nevertheless, there are still open questions as to whether cell type and hormone profile play a role in prognosis. Currently, the main factors assessed are grade based on proliferation (mitoses or Ki67 labeling index) and stage. However, the addition of hormone measurements, both preoperatively and in surveillance, associated with more sophisticated subtyping of these tumors based on transcription factors and hormone expression, will help to clarify this in future studies.

## Data Availability

Data is provided within the manuscript, figures and Table files.
